# DNA methylation changes in African American women with a history of preterm birth from the InterGEN study

**DOI:** 10.1186/s12863-021-00988-x

**Published:** 2021-09-05

**Authors:** Veronica Barcelona, Janitza L. Montalvo-Ortiz, Michelle L. Wright, Sheila T. Nagamatsu, Caitlin Dreisbach, Cindy A. Crusto, Yan V. Sun, Jacquelyn Y. Taylor

**Affiliations:** 1 School of Nursing, Columbia University, 560 W. 168th St, New York, NY 10032 USA; 2 Department of Psychiatry, Division of Human Genetics, School of Medicine, Errera Community Care Center-Orange Annex, Yale University, 200 Edison Road, Orange, CT 06477 USA; 3 School of Nursing & Dell Medical School, Department of Women’s Health, University of Texas at Austin, 1710 Red River St., Austin, TX 78712 USA; 4 Columbia University, Data Science Institute, Northwest Corner, 550 W 120th St #1401, New York, NY 10027 USA; 5 School of Medicine, Department of Psychiatry, Yale University, 389 Whitney Ave, New Haven, CT 06511 USA; 6 Rollins School of Public Health, Emory University, 1518 Clifton Road NE, Atlanta, GA 30322 USA; 7 Center for Research on People of Color, School of Nursing, Columbia University, 560 W 168th St, Room 605, New York, NY 10032 USA

**Keywords:** African American, Preterm birth, EWAS, DNA methylation

## Abstract

**Background:**

Preterm birth (< 37 weeks’ gestation) is a common outcome of pregnancy that has been associated with increased risk of cardiovascular disease for women later in life. Little is known about the physiologic mechanisms underlying this risk. To date, no studies have evaluated if differences in DNA methylation (DNAm) among women who experience preterm birth are short-term or if they persist and are associated with subsequent cardiovascular sequelae or other health disorders. The purpose of this study was to examine long-term epigenetic effects of preterm birth in African American mothers (*n* = 182) from the InterGEN Study (2014–2019). In this study, we determine if differences in DNAm exist between women who reported a preterm birth in the last 3–5 years compared to those who had full-term births by using two different approaches: epigenome-wide association study (EWAS) and genome-wide co-methylation analyses.

**Results:**

Though no significant CpG sites were identified using the EWAS approach, we did identify significant modules of co-methylation associated with preterm birth. Co-methylation analyses showed correlations with preterm birth in gene ontology and KEGG pathways. Functional annotation analysis revealed enrichment for pathways related to central nervous system and sensory perception. No association was observed between DNAm age and preterm birth, though larger samples are needed to confirm this further.

**Conclusions:**

We identified differentially methylated gene networks associated with preterm birth in African American women 3–5 years after birth, including pathways related to neurogenesis and sensory processing. More research is needed to understand better these associations and replicate them in an independent cohort. Further study should be done in this area to elucidate mechanisms linking preterm birth and later epigenomic changes that may contribute to the development of health disorders and maternal mood and well-being.

**Supplementary Information:**

The online version contains supplementary material available at 10.1186/s12863-021-00988-x.

## Background

Preterm birth, defined as the delivery of a neonate born prior to 37 weeks’ gestation [1], is a common outcome of pregnancy occurring in approximately 10% of births [2]. Neonatal consequences of preterm birth can be substantial including underdevelopment of major organs, respiratory distress, feeding difficulties, and developmental delays while also imposing significant financial and emotional burdens to families [3]. The burden of preterm birth is not equally distributed among people with the capacity for pregnancy, as African American women having the highest risk of preterm birth in the United States [1]. Preterm birth is the leading cause of infant morbidity and mortality, and African American women are 2.2 times more likely to have a baby born preterm than non-Hispanic White women, independent of maternal medical and socioeconomic variables [4, 5].

Preterm birth is often categorized as spontaneous or indicated. Spontaneous preterm birth refers to birth resulting from preterm labor, preterm spontaneous rupture of membranes, preterm premature rupture of membranes (PPROM) or cervical weakness. Indicated preterm birth occurs in response to maternal or fetal distress. The etiology of spontaneous preterm birth is multifactorial and often difficult to causally determine for an individual patient. Factors such as maternal infection, premature rupture of membranes, and medically indicated induction for pre-eclampsia or intrauterine growth restriction are common reasons for preterm birth [6]. While the biological underpinnings of preterm birth are unclear, genetic factors have been proposed. Genome-wide association studies (GWAS) have identified replicable, robust associations of six genomic loci [7] and over 100 candidate gene polymorphisms with potential functional relevance to preterm birth [8]. These genetic loci could be potential targets for developing interventions, however they only explain a small proportion of the variance associated with preterm birth. Emerging technology can now allow us to investigate how the combinatorial effect of the environment may also be responsible.

Epigenetics, the interplay between genetic and environmental factors, have been a generally understudied area to understand the long-term outcomes of preterm birth [9]. More specifically, limited evidence has focused on the epigenetic changes associated with preterm birth in African Americans despite the known intergenerational, environmental, psychological, and physiological stressors [10]. The effect of stressors as a result of preterm birth, such as potential financial instability due to increased medical needs, neonatal intensive care, and variable social support have not been thoroughly examined on a mother over her life course. This is an example of a serious pregnancy-related concern that disproportionately places attention on the neonatal in comparison to the health and wellbeing of the mother. Recent advances in research equity have placed further emphasis on the need to evaluate durable effects to women, not just her child.

Preterm birth and other pregnancy complications have been associated with increased risk of cardiovascular disease later in life [11, 12], independent of additional cardiovascular risk factors [13, 14]. Some have postulated that this may be because pregnancy is a “stress-test” or window for future cardiovascular risk [15], yet little is known about the physiologic mechanisms underlying this hypothesis. Epigenome-wide association studies (EWAS) of preterm birth have historically been conducted with the intent to identify potential risk factors or biomarkers for preterm birth [16]. Most studies on the epigenomics of preterm birth have examined women during pregnancy, and have not focused on the later effects on women years after birth. To date, no studies have evaluated if differences in DNA methylation (DNAm) among women who experience preterm birth are short-term or if they persist and are associated with subsequent cardiovascular sequelae or other health disorders.

The purpose of this study was to examine the long-term epigenetic effects of preterm birth on African American mothers. In this study, we determine if differences in DNAm exist between women who reported a preterm birth in the last 3–5 years compared to those who had full-term births by using two different approaches: EWAS and genome-wide co-methylation analysis.

## Methods

The Intergenerational Impact of Genetic and Psychological Factors on Blood Pressure (InterGEN) Study was conducted between 2014 and 2019 in Connecticut. Mothers (*n* = 250) enrolled with a biological child aged 3–5 years old (*n* = 250) for this longitudinal study (*N* = 500). Women were eligible to participate in the study if they were ≥ 21 years old, self-identified as African American or Black, spoke English, had no mental illness that could interfere with reliable response to self-reported psychological measures, and enrolled with a biological child (3–5 years old). There was a total of four study visits, each 6 months apart. Baseline data were collected at the Time (T) 1 visit, including demographic information, smoking status, clinical measurements (height, weight, and blood pressure), and salivary DNA. The purpose of the InterGEN study was to examine Gene (G)- Environment (E) interactions on blood pressure for mothers and children. DNAm of genes associated with blood pressure (G) and environmental factors (E) (racism/discrimination, parenting stress, and maternal depression) were studied. A diagnosis of high blood pressure was not a requirement for study participation. Audio Computer Assisted Self-Interview (ACASI) software was used for self-reported data collection. Mothers reported gestational age at birth for the enrolled child at the T2 interview by answering “How many weeks pregnant were you when your (enrolled) child was born?” They indicated length of gestation by choosing from a list ranging from 20 to 42 weeks. Preterm births were categorized as those occurring before 37 weeks gestation. A sample (*n* = 77) of the responses for gestational age were objectively validated by medical record abstraction. Recruitment, psychological measures, and genetic methods are discussed elsewhere [17, 18]. Institutional Review Board approval was received from the associated institutions; and written, informed consent was obtained from all participants. All methods were carried out in accordance with relevant guidelines and regulations.

Passive saliva was collected in Oragene (OG)-500 format tubes according to established study protocols. Saliva samples were transported to the research laboratory and refrigerated at 4 °C until processed. DNA extraction and purification were carried out using ReliaPrep kits, and the Illumina Infinium Methylation EPIC (850 K) BeadChip was used for epigenome-wide DNAm measurement. Sample processing and methylation analyses were conducted at the Yale Center for Genome Analysis. The resulting genetic data contained methylated (M) and unmethylated (U) signals used to calculate β values, where β = M/(M + U) and varying from 0.0 to 1.0.

### Epigenomic statistical analysis

Quality control was performed using the ‘minfi’ R package (version 1.32.0) [19]. Subsequently, we filtered out cross-reactive probes, probes with detection *p*-value > 0.001, and located in sex chromosomes. The batch effect correction was conducted using the ComBat method from the ‘sva’ R package (version 3.34.0) [20]. Normalization was performed using the functional normalization method in the ‘minfi’ R package. The β values were used to conduct a cell composition estimation analysis (CD14, CD34, and buccal cells) according to the Houseman method [21]. Principal component analysis (PCA) was conducted to identify population stratification using the Barfield method [22]. After quality control, 846,459 sites were retained and included in EWAS analysis.

Association analysis to detect differentially methylated CpGs associated with preterm birth was conducted using the ‘cpg.assoc’ function from the ‘minfi’ R package. The linear regression model examined the association between preterm birth and DNAm, adjusting for maternal age, cell proportions (CD14, CD34, and buccal cells), the top three principal components (PCs), and smoking status. Multiple testing correction was conducted using False Discovery Rate (FDR) of less than 0.05.

### Co-methylation analysis

Co-methylation analysis was performed using the ‘WGCNA’ R package (Version 1.69) [23]. The network construction was set to a soft-thresholding power of 3 and ‘minModuleSize’ equal to 30 using the function ‘blockwiseModules’. The analysis clustered the correlated differentially methylated sites into modules, calculating a value to represent the methylation profile for each module (eigengene). Using the eigengene, we calculated a module correlation and a *p*-value for the association with preterm birth. Genes within modules with correlation ≥ |0.13| and *p*-value **≤** 0.05 were selected for functional annotation and interactome analysis. The gene significance and module membership were evaluated for the two modules with biological significance.

### Functional annotation analysis

The CpG annotation for the EWAS and co-methylation analysis was conducted using the ‘IlluminaHumanMethylationEPICanno.ilm10b4.hg19’ R package (Version 0.6.0) [24]. Functional annotation analysis was conducted using the ‘gometh’ function from ‘missMethyl’ R package (Version 1.20.4) [25]. Significant Gene Ontology (GO) and Kyoto Encyclopedia of Genes and Genomes (KEGG) enrichment was defined as FDR threshold of 0.05.

### Interactome

The interactome analysis was performed with the STRING database (Version 11.0) [26]. We set a confidence threshold of 0.9 and select databases from co-expression analysis, high-throughput experiments, experimental information, and databases. The database uses previous information to calculate a score for each interaction, and network edges refer to the confidence of the interaction.

### DNA methylation age

DNAm age acceleration is defined as the residual term of a univariate model regressing estimated DNAm age on maternal chronological age. We used 353 age-related biomarkers identified by Horvath [27–29]. Horvath’s is the only cross-tissue method that is valid for examination of saliva samples, as most studies examine multiple tissues for DNAm analysis. We conducted a linear regression analysis to evaluate the association between DNAm age acceleration and preterm birth. DNAm age acceleration was modeled with independent variables of preterm birth, age, smoking status, and hypertension status. Analysis was conducted using R Studio (Version 1.3.959) with R version 3.4.3.

## Results

A total of 250 women were enrolled at the T1 InterGEN visit, however, only 191 completed the T2 visit where preterm birth status of the enrolled child was assessed. Participants were excluded from analyses if they: did not complete the T2 visit or were missing data on preterm birth (the primary exposure) (*n* = 64), had a multiple gestation (*n* = 2), and if they had missing/insufficient DNA (primary outcome) for analysis (*n* = 2). This resulted in a final sample of 182 women contributing data for the present analysis. Participant characteristics are summarized in Table 1. Most women were between the ages of 30–39 (50.5%), had completed some college or more education (61.2%), and reported an annual income of less than $15,000 (45.1%). Women were most commonly insured by Medicaid (60.7%), were obese (body mass index ≥30 kg/m^2^) (46.1%) and were non-smokers (78.3%). Approximately a quarter of women (25.2%) had a preterm birth with their enrolled child. Validation of a subsample of preterm births was completed using medical record abstraction, and self-report was found to be highly correlated to objectively ascertained gestational age.

**Table 1:** Participant characteristics, InterGEN Study, 2017–2020, *n* = 182

	Term birth (≥37 weeks)	Preterm birth (< 37 weeks)	Total n (%)
Age			
20–29	58	16	74 (40.6)
30–39	66	26	92 (50.5)
40–49	12	4	16 (8.7)
Highest education completed			
< High School	5	4	9 (4.9)
High School graduate	43	18	61 (33.7)
Some college	48	14	62 (34.2)
≥ Associate degree/College graduate	40	9	49 (27.0)
Annual household income			
≤ $15,000	60	19	79 (45.1)
> $15,000–$50,000	55	22	77 (44.0)
≥ $50,000	17	2	19 (10.8)
Health insurance			
Private/employer	21	6	27 (14.9)
Medicaid	81	29	110 (60.7)
Government/ACA	21	6	27 (14.9)
Other	5	2	7 (3.8)
Uninsured	8	2	10 (5.5)
Body mass index (BMI)			
Underweight (< 18.5)	3	4	7 (3.8)
Normal weight (18.5–24.9)	36	8	44 (24.1)
Overweight (25–29.9)	35	12	47 (25.8)
Obese (≥30)	62	22	84 (46.1)
Current smoker			
No	111	30	141 (78.3)
Yes	25	14	39 (21.6)

### Epigenome-wide association analysis

The Manhattan plot depicting the association between DNAm and preterm birth in African American women is presented in Supplemental Figure 1. The quantile-quantile plot is presented in Supplemental Figure 2 with a lambda value of 1.06. After multiple testing correction, no epigenome-wide significant CpG sites associated with preterm birth were identified, and the top 15 associations are presented in Table 2. The top finding associated with preterm birth was cg02083539 (*p* = 2.08 × 10^− 6^, FDR = 0.51) mapped to the *DOC2A* gene. Functional annotation of the top 500 CpG sites associated with preterm birth found no significant GO and KEGG enrichment at FDR < 0.05. The top three nominal associations for GO pathways include vasopressin receptor binding, maternal aggression, and positive regulation for cellular pH reduction. The top three nominal associations for the KEGG pathways include vascular smooth muscle contraction, cGMP-PKG signaling pathway, and vasopressin-regulated water reabsorption.

**Table 2:** Top 15 differentially methylated CpG sites associated with preterm birth, InterGEN

CpG site	Gene Name	Chr	Position	Promoter Associated	FDR	*P*-value
cg02083539	*DOC2A*	16	30,022,586	NO	0.51	2.08E-06
cg14696870	*FCER1A*	1	159,258,877	NO	0.51	3.00E-06
cg19365615	*NQO2*	6	2,999,313	YES	0.51	3.67E-06
cg16492510		7	157,561,684	NO	0.68	6.29E-06
cg06765321	*GMCL1*	2	70,057,479	YES	0.75	1.05E-05
cg24049621	*ZNF532*	18	56,528,679	NO	0.75	1.07E-05
cg13372811	*RP4-773 N10.6*	1	110,627,590	NO	0.75	1.18E-05
cg08578323	*NDUFAF2*	5	60,428,301	NO	0.77	1.36E-05
cg02437376	*VDAC2*	10	76,969,880	NO	0.8	1.61E-05
cg25270236	*U2*	10	27,482,721	NO	0.8	1.85E-05
cg15352013		16	49,497,733	YES	0.8	2.04E-05
cg06473773	*SNORA71D*	20	37,063,729	YES	0.8	2.39E-05
cg01078147	*SEMA6A*	5	115,911,468	NO	0.8	2.49E-05
cg27478704	*TMEM63C*	14	77,662,756	NO	0.8	2.51E-05
cg07215395	*CDO1*	5	115,148,221	NO	0.8	2.56E-05

### Co-methylation analysis

**Fig. 1:**
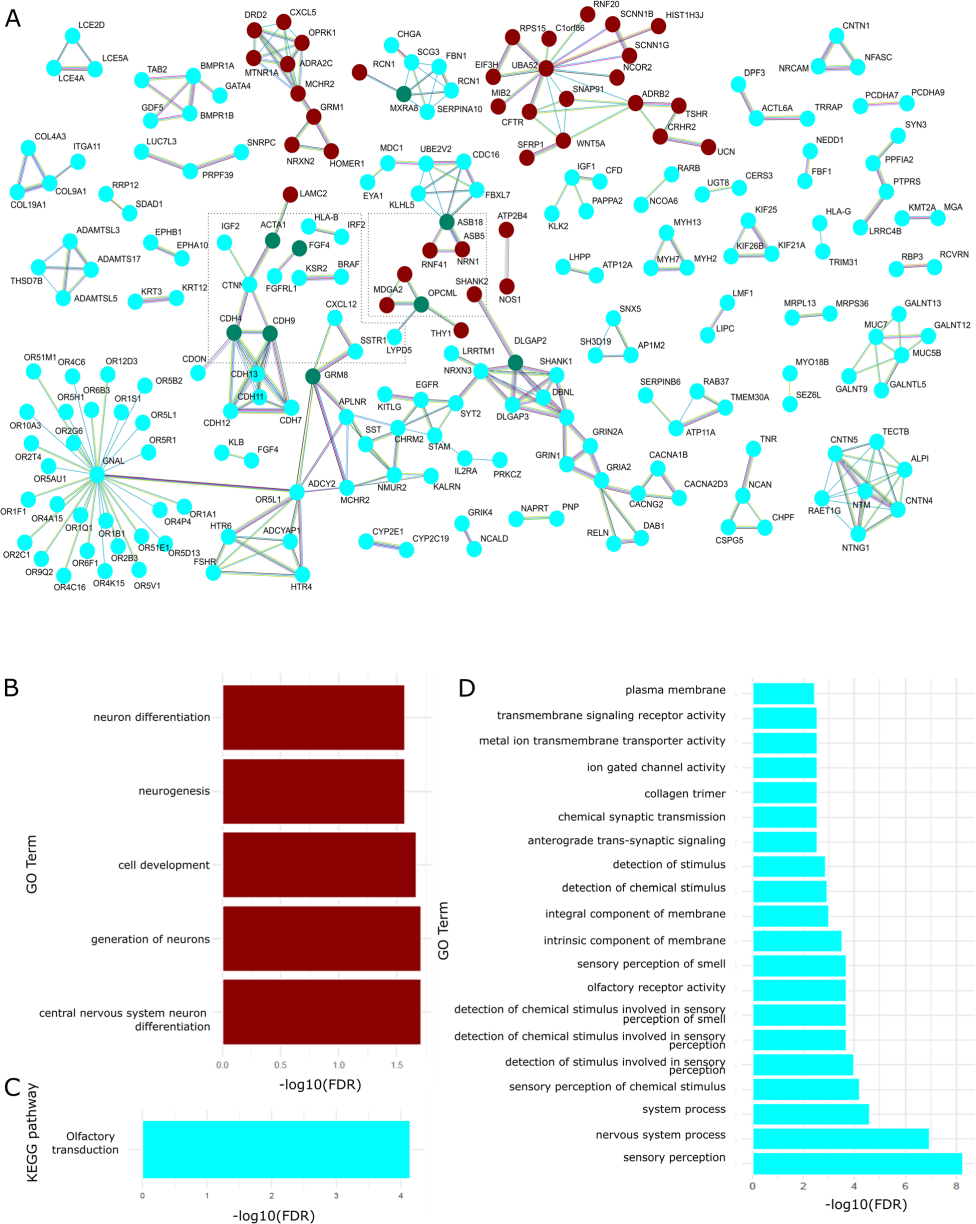
Functional annotation and protein-protein interaction analyses. **A** Networking to cyan and darkred modules. The modules were created separately to each sub-group: positive and negative correlation with preterm birth in each module. The green nodules identify proteins detected in more than one subgroup. **B** GO enrichment analysis to darkred module. **C** KEGG enrichment analysis to cyan module. **D** GO enrichment analysis to cyan module

A total of 302 significant modules were identified, of which 45 had a correlation ≥|0.13| and *p*-value < 0.05. Functional annotation for all the 45 modules showed two modules enriched for GO terms (cyan and darkred) and one for KEGG pathways (cyan). Functional annotation and protein-protein interaction (PPI) analysis are depicted in Fig. 1.

The darkred module was identified with a positive correlation with preterm birth (corr = 0.14, *p*-value = 0.04) and age (corr = 0.17, *p*-value = 0.01). The GO enrichment (Fig. 1b) detected genes involved in cell development, generation of neurons, and central nervous system development. PPI analysis was conducted on genes stratified by positive and negative correlation with preterm birth. The majority of the genes in the darkred module were positively correlated with preterm birth and enriched for transcription DNA-binding transcription factor activity (FDR 5.15 × 10^− 5^). Negatively correlated sites did not show significant GO enrichment. PPI analysis (Fig. 1a) of positively correlated CpGs into the darkred module did not show enrichment to all GO terms observed in the whole module.

The cyan module showed a negative correlation with preterm birth (corr = − 0.14, *p*-value = 0.03). For this module, we did not identify a correlation with age or smoking. The functional annotation to the cyan module identified olfactory transduction pathway enriched in the KEGG analysis (Fig. 1c) and metal ion transmembrane transporter activity, collagen trimer, chemical synaptic transmission, sensory perception of smell, and olfactory receptor activity in the GO analysis (Fig. 1d). When stratifying CpGs into those positively and negatively correlated with preterm birth, we identified enriched terms involved in metal ion binding (FDR = 0.04) and transcription regulator activity (FDR = 0.04) in the positively correlated subgroup. The negatively correlated subgroup showed KEGG enrichment to the olfactory transduction pathway (FDR = 0.00051) and GO enrichment to calcium ion binding (FDR = 6.53 × 10^− 6^), olfactory receptor activity (FDR = 0.022), and transmembrane signaling receptor activity (FDR = 0.031). In the negative subgroup of cyan modules, we found the same GO terms that those detected in the whole module.

We also detected an overlap of the genes in PPI analysis to the subgroups from darkred and cyan modules (green nodes in Fig. 1a). This indicates that these two modules show a different pattern of correlated methylation.

### DNA methylation age

**Fig. 2:**
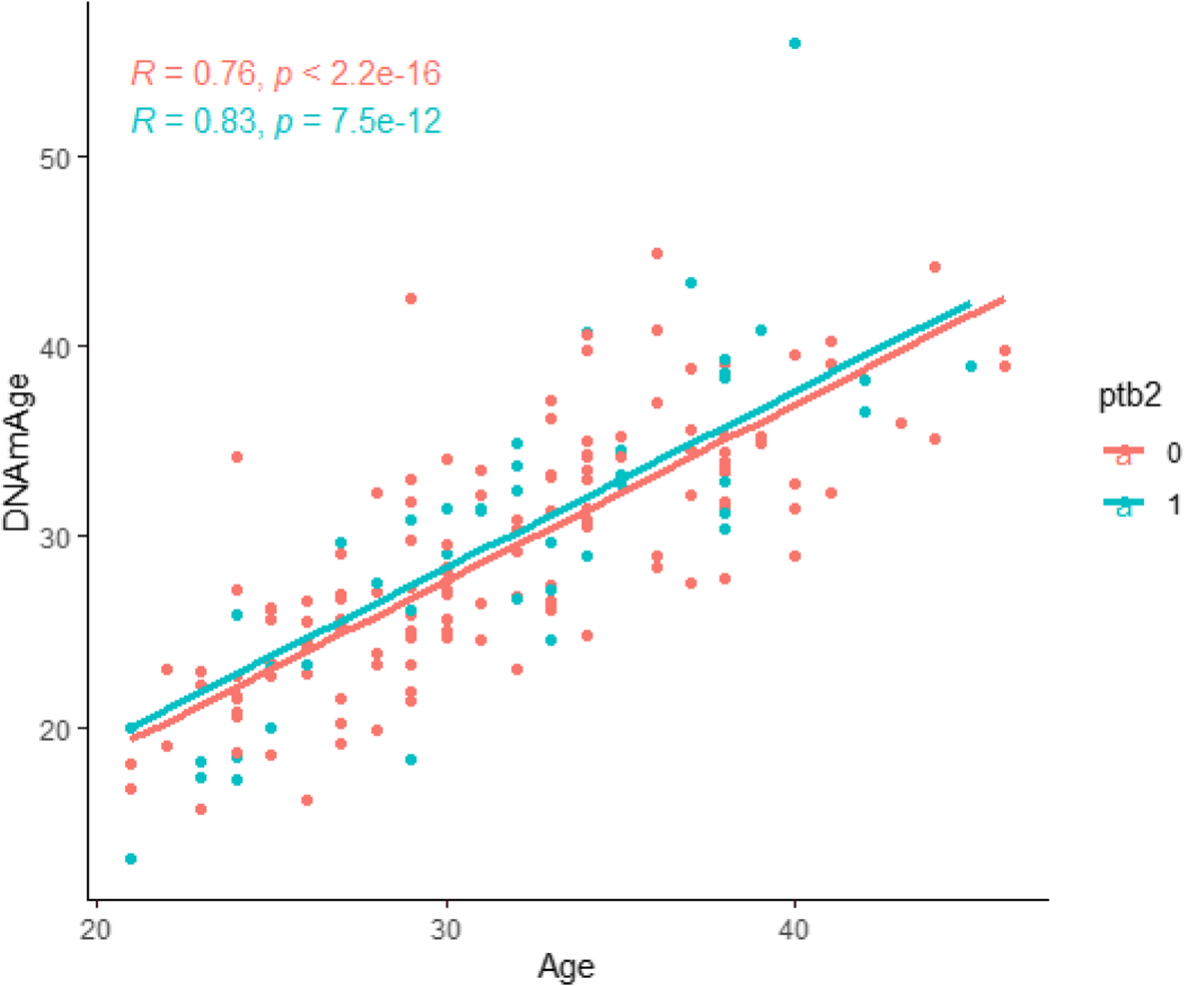
Association of maternal chronological age (x-axis) and DNAm age (y-axis) by preterm birth status in the InterGEN study. The samples were stratified by full-term (0) and preterm birth (1)

We observed a strong correlation between DNAm age and chronological in the InterGEN cohort. There was a significant association between hypertension and DNAm age; however, no association was identified between DNAm age and preterm birth (Table 3). Figure 2 shows a high correlation between chronological age and DNAm age for women in both control (*R* = 0.76, *p* < 2.2 × 10^− 16^) and preterm birth (*R* = 0.83, *p* = 7.5 × 10^− 12^) groups.

**Table 3:** Associations for DNAm age analyses

	Estimated 훽	Std. Error	t value	Pr(>|t|)
Maternal age	0.89162	0.05854	15.231	< 2 × 10^16^
Preterm Birth	0.53871	0.76782	0.702	0.4839
Smoking	−1.17955	0.79408	−1.485	0.1393
Hypertension	1. 71,089	0.82859	2.065	0.0405

## Discussion

In this study, we identified epigenetic changes associated with preterm birth history among African American women 3–5 years after delivery. Though no significant CpG sites were identified using the EWAS approach, we did identify significant modules of co-methylation associated with preterm birth. Co-methylation analyses showed a negative correlation of darkred with preterm birth, and a positive correlation with the cyan module. Functional annotation analysis revealed enrichment for pathways related to central nervous system and sensory perception. No association was observed between DNAm age and preterm birth, though larger samples are needed to confirm this further.

Previous studies have examined the epigenomic effects of preterm birth on both mothers and their newborns, however, none have focused on the epigenetic effects on the mother after having a child born preterm [30–32]. Wang et al. [30] reported DNAm changes in placental tissue and cord blood of newborns born preterm compared to term infants in China (*n* = 48). These changes were localized in genes associated with cellular regulation and metabolic processes. Another study carried out in an Asian cohort (*N* = 1019) of term and preterm infants reported differential methylation in cord tissue and blood among infants born preterm, in sites associated with fetal growth and development, and immune response pathways, respectively [31]. One study investigated preterm birth and DNAm among African Americans [32], examining maternal peripheral blood among women who delivered preterm (*n* = 16) and at term (*n* = 24). They identified differential methylation of genes associated with metabolic, cardiovascular and immune pathways among women who delivered preterm compared to those who delivered at term.

In this study, we identified several co-methylation modules associated with preterm birth. The darkred module showed enrichment for cell development, neurogenesis, and central nervous system. The cyan module was enriched for olfactory signaling, sensory perception, synaptic transmission, calcium ion binding, and transcription regulation. Interestingly, previous research has found women with preterm birth history are at increased risk for cardiovascular disease, mood disorders, and perceptions of their baby [33]. Little is known about how epigenetic changes associated with pregnancy or preterm birth may relate to sensory perception and central nervous system functioning, years after birth. A single review was identified which studied olfactory sensitivity in pregnancy, and the authors noted that more research is needed to support anecdotal data and the relationship to hormonal changes of pregnancy [34]. Olfaction has been associated with maternal-infant bonding [35], which is a process that may be interrupted or delayed in preterm births. This area of research may benefit from the inclusion of epigenetics to examine potential long-term effects on cellular functioning in mothers. More research is needed to better understand the mechanisms and relationship between identified biological pathways from the co-methylation analysis and the effects of preterm history on the mother, as well as the direction of effects observed.

Strengths of this study include that this is a secondary analysis of data from a cohort study comprised of African American women, a group that is underrepresented in epigenomic studies, as well as a focus on the understudied topic of effects of preterm birth on mothers in the 3 to 5-year time period after delivery. Limitations include the potential for residual confounding, cross-sectional design, and lack of baseline DNAm levels for women in the perinatal period. The rate of preterm birth reported by women in our study was higher than reported in nationally representative data for African Americans [36], and this may be due to targeted recruitment of participants in urban, low income neighborhoods. Another potential explanation is the self-reported preterm birth ascertainment as objective birth outcome data was not available for all participants. Maternal self-report of gestational age, however, has been found to be a valid method of assessment and comparable to medical record review [37]. Despite this, we acknowledge the imprecision of our measurement 3–5 years after birth and the risk of residual confounding. As this was a secondary analysis of existing data, we did not have information on other preterm births that mothers may have had in their lifetime, nor did we have data on the specific phenotypes of preterm birth (i.e. spontaneous or elective). Our findings may be affected by selection bias as 35% of eligible mothers approached for participation in InterGEN were enrolled. Demographic data were not available to compare enrolled women to those who were not enrolled and rule out this possibility. Our study differs from previous work for a variety of reasons. Previous studies have focused on the immediate perinatal period, and most studies were conducted on children. DNA for the present study was extracted from saliva, while others used cord blood, cord tissue, or peripheral blood. Most of the previous studies have not included African Americans, and sample sizes have varied greatly.

## Conclusion

In summary, we identified differentially methylated gene networks associated with preterm birth in African American women 3–5 years after birth, including pathways related to neurogenesis and sensory processing. More research is needed to understand better these associations and replicate them in an independent cohort. Further study should be done in this area to elucidate mechanisms linking preterm birth and later epigenomic changes that may contribute to the development of health disorders and maternal mood and well-being.

## Supplementary Information

**Additional file 1: Supplemental Figure 1.** Manhattan Plot of epigenome-wide associations with preterm birth, InterGEN. **Supplemental Figure 2.** Quantile-Quantile Plot for association between DNA methylation and preterm birth, InterGEN.

## Data Availability

The datasets analyzed in the current study are available on dbGaP- accession: phs001792.v1.p1.

## Abbreviations

DNA: Deoxyribonucleic Acid
DNAm: DNA methylation
PPI: Protein- Protein Interaction
GO: Gene Ontology
KEGG: Kyoto Encyclopedia of Genes and Genomes
FDR: False Discovery Rate
CpG: 5′—C—phosphate—G—3
cGMP-PKG: Cyclic Guanosine Monophosphate- Protein Kinase G
InterGEN: The Intergenerational Impact of Genetic and Psychological Factors on Blood Pressure Study
T1-T4: Time 1-Time 4
PC: Principal Components
PCA: Principal Components Analysis
ACASI: Audio Computer Assisted Self-Interview
EWAS: Epigenome-Wide Association Study
GWAS: Genome-Wide Association Study
PPROM: Preterm Premature Rupture Of Membranes
