# Supplementary material for: DNA methylation changes in African American women with a history of preterm birth from the InterGEN study
Source: BMC Genom Data. 2021 Sep 5;22:30. doi: 10.1186/s12863-021-00988-x (PMC8418749; doi:10.1186/s12863-021-00988-x)

**Supplemental Figures**

Supplemental Figure 1. Manhattan Plot of epigenome-wide associations with preterm birth, InterGEN.


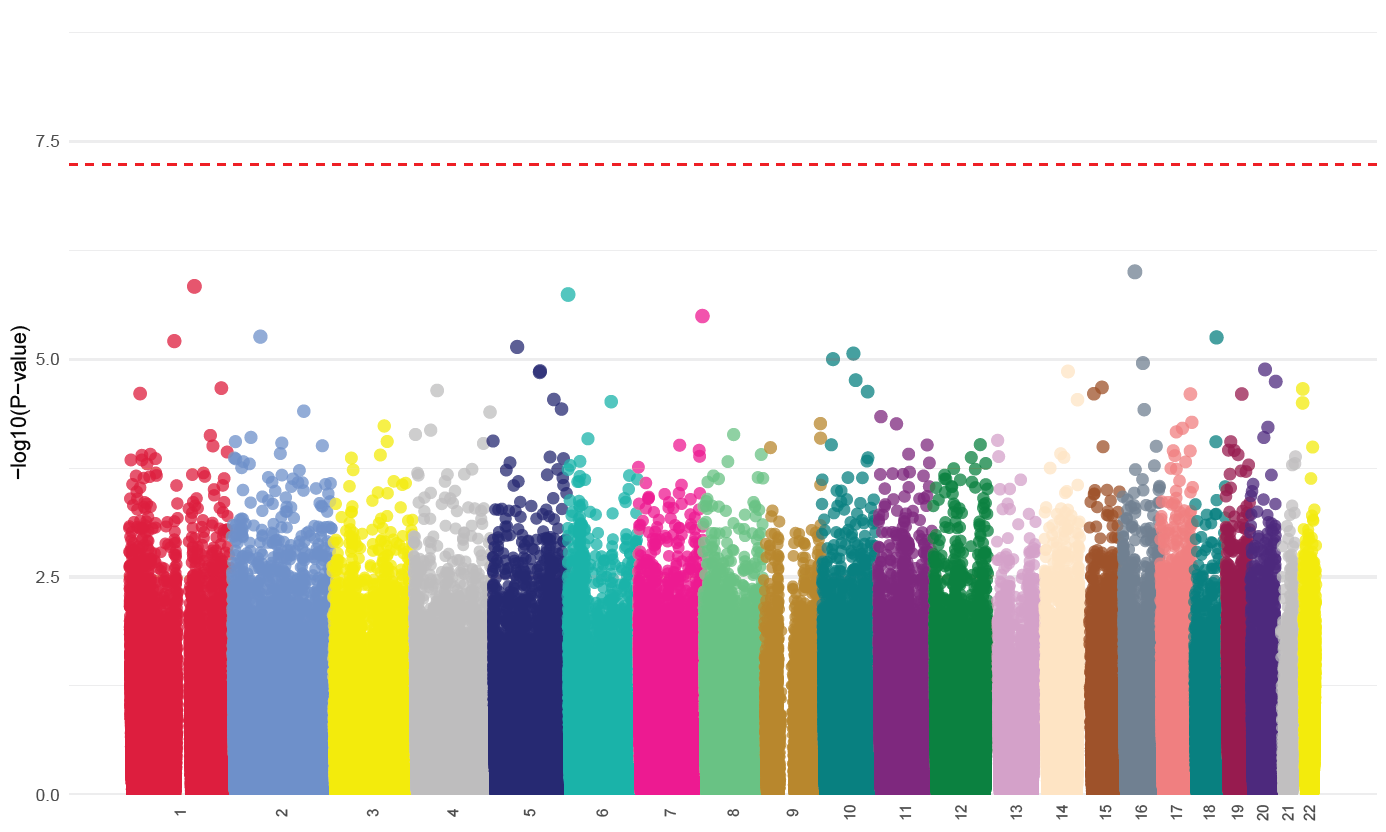


Supplemental Figure 2.

Quantile-Quantile Plot for association between DNA methylation and preterm birth, InterGEN.


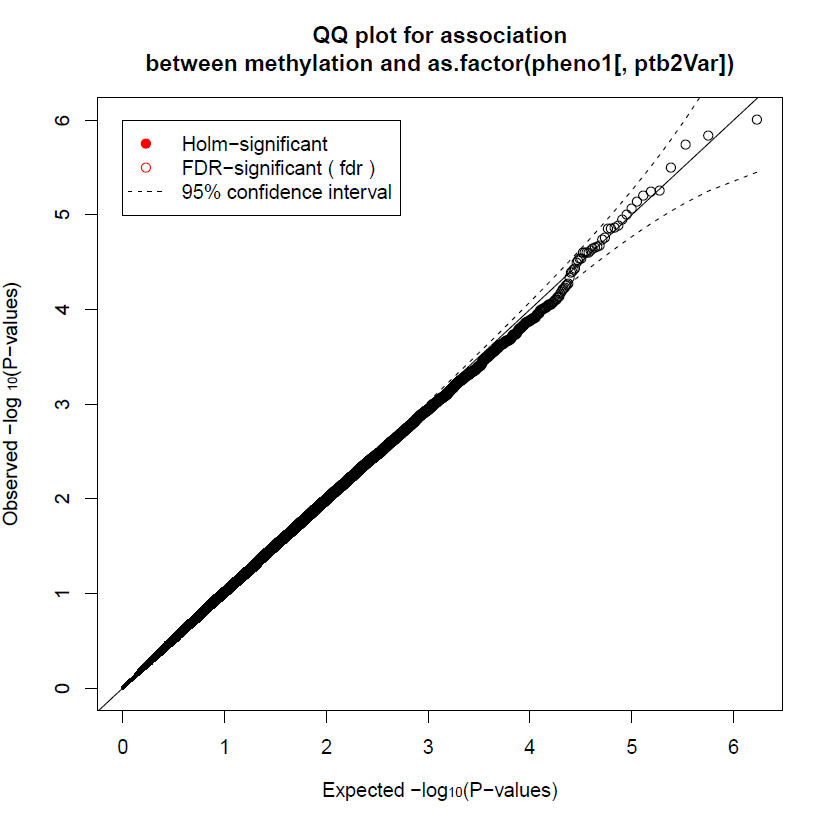

Supplement: Supplementary file 1 — Additional file 1: Supplemental Figure 1. Manhattan Plot of epigenome-wide associations with preterm birth, InterGEN. Supplemental Figure 2. Quantile-Quantile Plot for association between DNA methylation and preterm birth, InterGEN. [file 12863_2021_988_MOESM1_ESM.docx]
